# Study on the Association of Dietary Fatty Acid Intake and Serum Lipid Profiles With Cognition in Aged Subjects With Type 2 Diabetes Mellitus

**DOI:** 10.3389/fnagi.2022.846132

**Published:** 2022-03-31

**Authors:** Pengfei Li, Yanyan Gao, Xiaojun Ma, Shaobo Zhou, Yujie Guo, Jingjing Xu, Xixiang Wang, Nicholas Van Halm-Lutterodt, Linhong Yuan

**Affiliations:** ^1^School of Public Health, Capital Medical University, Beijing, China; ^2^School of Life Sciences, Institute of Biomedical and Environmental Science and Technology, University of Bedfordshire, Luton, United Kingdom; ^3^School of Professional Studies, Brown University, Providence, RI, United States

**Keywords:** type 2 diabetes mellitus, fatty acids, lipid, cognition, older adults

## Abstract

**Background:**

The correlation between dietary fatty acid (FA) intake and serum lipid profile levels with cognition in the aged population has been reported by previous studies. However, the association of dietary FA intake and serum lipid profile levels with cognition in subjects with type 2 diabetes mellitus (T2DM) is seldom reported.

**Objective:**

A cross-sectional study was conducted to explore the correlation between dietary FA intake and serum lipid profiles with cognition in the aged Chinese population with T2DM.

**Methods:**

A total of 1,526 aged Chinese subjects were recruited from communities. Fasting blood samples were collected for parameter measurement. The food frequency questionnaire (FFQ) method was applied for a dietary survey. Cognition was assessed using the Montreal Cognitive Assessment (MoCA) test. Dietary FA intake and serum lipid levels were compared between subjects with T2DM and control subjects. A logistic regression analysis was carried out for analyzing the association of FA intake and serum lipid levels with the risk of mild cognitive impairment (MCI) in subjects with T2DM and control subjects.

**Results:**

There was a significant difference in the serum lipid level between the T2DM group and the control group. Results of the logistic regression analysis demonstrated the potential associations of serum total cholesterol (TC), low-density lipoprotein cholesterol (LDL-c), and dietary n-3 polyunsaturated fatty acids (PUFAs) intake with the risk of MCI in subjects with T2DM, but the associations were not observed in control subjects.

**Conclusion:**

The T2DM phenotype might affect the relationship between dietary FA intake, circulating lipids, and cognitive performance. Large prospective cohort studies are needed to uncover the underlying mechanism of how dietary FA intake and serum lipid levels affect cognition in aged subjects with T2DM.

## Introduction

Alzheimer’s disease (AD) is the leading cause of dementia in light of its greater prevalence and contributes to approximately 70% of all dementia cases (Reitz and Mayeux, 2014). By 2030, the proportion of older adults aged 60 years and above is estimated to rise currently from 15 to 25% in China. With the rapid increase in the aged population in China, the number of patients with AD is expected to substantially increase in the near future (Yee et al., 2018). However, there is no sufficient method for the clinical treatment of AD. Therefore, the identification of potential risk factors and the application of precise prevention and intervention of AD based on those risk factors have become an urgent global public concern.

Epidemiological studies demonstrated the close relationship between type 2 diabetes mellitus (T2DM) and AD (Asih et al., 2017), e.g., a patient with T2DM exhibits 2.0− to 2.5-fold risk of AD than healthy subjects (Kandimalla et al., 2017). Moreover, hyperglycemia was found to correlate with poor cognitive performance and to accelerate progression of cognitive impairment to dementia (Pugazhenthi et al., 2017). Population-based studies observed that a diabetic phenotype might exacerbate cognitive impairment and brain atrophy in patients with AD (Shinohara and Sato, 2017). Results of animal experiments indicate that T2DM and AD shared similar pathophysiological processes, such as metabolic disorder and damage of insulin pathways (Miklossy and McGeer, 2016); thus, some researchers colloquially termed AD as the type 3 diabetes mellitus. Several mechanisms including hyperinsulinemia, oxidative stress, insulin resistance, advanced glycation end products, and overt immune system activation were contributable to the linkage of AD and diabetes (Baglietto-Vargas et al., 2016). The putative “shared pathways” in AD and T2DM imply the great potential of antidiabetes strategies in the prevention and treatment of AD in the aged population with T2DM (Karki et al., 2017).

Dyslipidemia is a major phenotype of diabetes mellitus (DM), which is characterized as hypertriglyceridemia, high-density lipoprotein (HDL) hypocholesterolemia, and low-density lipoprotein (LDL) hypercholesterolemia (Mooradian, 2009). Correlations of dietary fatty acid (FA) intake with DM have caused extensive attention. A case-cohort study demonstrated that circulating even-chain saturated fatty acids (SFAs) are positively associated with the incidence of T2DM, while a negative association was observed for odd-chain SFAs (Forouhi et al., 2014). However, a meta-analysis indicated null association of SFAs with T2DM (de Souza et al., 2015). Polyunsaturated fatty acids (PUFAs) supplements were found to relieve T2DM phenotypes (Zubrzycki et al., 2018). On the contrary, other studies found that *n*-3 PUFAs and its ratio to *n*-6 PUFAs (*n*-3/*n*-6) showed no effects on the glucose metabolism in subjects with DM (Brown et al., 2019), and high PUFAs intake was associated with an increased risk of T2DM (Guasch-Ferré et al., 2017). Despite these diversities of results from different studies, a growing number of evidence suggests the role of FAs in affecting T2DM phenotypes. In view of the potential association of T2DM with cognition, it is necessary to explore the relationship between dietary FA intake, serum lipid levels, and their effects on cognition in aged subjects with T2DM.

The relationship between lipids and cognition has been reported by the previous study (Shin et al., 2018). Additionally, deficits of executive function and memory abilities were reportedly associated with hypercholesterolemia (Suárez Bagnasco, 2017). A recent evidence showed that diet-induced dyslipidemia could significantly affect the neuromotor activity, the anxiety level, and cognitive functions in rats and mice (Apryatin et al., 2017). Meanwhile, animal experiments also showed that the high-fat diet accelerates age-associated cognitive decline *via* harming the blood-brain barrier, upregulating brain amyloid beta 1-40 (Aβ_1–40_) accumulation and deteriorating cerebral oxidative stress (Thériault et al., 2016). On the contrary, a low-fat diet could cause pronounced weight loss, decreased inflammatory response, and improved glucose intolerance in the wild-type and AD mice models (Walker et al., 2017). Dietary PUFAs, especially *n*-3 PUFAs, may improve cognitive performance in older people with mild cognitive impairment (MCI) by enhancing cerebral blood flow, reducing inflammation, and mitigating amyloid plaque formation and aggregation (Zhang et al., 2020). However, other studies have not found any conclusive evidence of the association between dietary fat intake, plasma lipid profiles, and cognition in patients with T2DM (Li et al., 2018).

Given the rapid increase of the aged population and a high incidence of T2DM in China, we, therefore, conducted this study aiming to explore the correlation between dietary FA intake and serum lipid profiles with cognition in aged subjects with T2DM. Our data provide basic information for revealing the etiology of diabetes-related cognitive impairment in older adults.

## Materials and Methods

### Participants

A total of 1,751 subjects aged 50–75 years were recruited from the Nanyuan and the Wulituo communities in Beijing. Subjects with the following status were excluded from the investigation: those suffering from dementia or Parkinson’s disease; those with a history of acute cerebrovascular disease; with active epilepsy; those with severe sensory perceptive disorders and who could not accomplish cognition measures and dietary survey; those suffering from other severe or unstable medical diseases likely affecting the assessment of brain functions or cognitions; and those with psychological history with depression, mania, delirium, or anxiety. Diabetes was ascertained according to the guidelines for the prevention and control of T2DM in China (CDS, 2018) (2017 Edition, Chinese Diabetes Society). The study protocol conforms to the ethical guidelines of the 1975 Declaration of Helsinki. The studies involving human participants were reviewed and approved by The Medical Ethics Committee of Capital Medical University (No. 2012SY23). The patients/participants provided their written informed consent to participate in this study.

### Demographic Characteristics and Anthropometric Measures

Demographic characteristics including age; gender (i.e., male or female); body mass index (BMI); education; drinking alcohol (i.e., yes/no); smoking (i.e., no, abandon tobacco, and current smoking); physical activity (i.e., never, 1–3 days/week, 4–6 days/week, and every day); reading habit (i.e., yes/no); watching TV and using the computer (yes/no); living status (i.e., living alone: yes/no); history of a disease, such as hyperlipidemia (i.e., yes/no), cerebrovascular accident (i.e., CVA, yes/no), and chronic kidney disease (CKD) (i.e., yes/no); eating fish oil supplements (i.e., yes/no); and AD family history (i.e., yes/no) were collected by the self-administered questionnaire. Education level was evaluated according to the highest level attained and was classified into six categories (i.e., illiterate, primary school, junior high school, high school, junior college, and undergraduate and above). Anthropometric parameters (e.g., height and weight) were measured by nurses in the health center of the community. BMI was calculated as weight in kilograms divided by height in the square of height in meters.

### Dietary Investigation

A validated semi-quantitative food frequency questionnaire (FFQ) was used to investigate dietary intake. This questionnaire was designed according to the version adopted by the Chinese Nutrition Society. The consumption frequencies (i.e., daily and weekly) and the intake amounts of 11 food items were investigated face to face by specifically trained nutritionists and registered nurses. The food-item list included cereal, fruits, vegetables, beans, cooking oil, fish, red meat, poultry meat, nuts, milk, and egg. The intake of cooking oil, especially, was calculated averagely in line with the number of dining individuals of each family and the monthly consumed amount according to the previous description (Zhang et al., 2009). Daily intake of FAs (e.g., SFAs, MUFAs, PUFAs, *n*-6 PUFAs, *n*-3 PUFAs, and *n*-6/*n*-3 PUFAs ratio) was calculated according to the Chinese Food Composition (2016) (Yang, 2016).

### Cognition Assessment

The Montreal cognitive assessment (MoCA) test was applied to evaluate the cognitive function of the participants by trained investigators. The MoCA test consisted of seven cognitive domains, including visual and executive ability, naming, attention, language, abstraction, memory and delayed recall, and orientation. This method was a cognitive screening tool with high sensitivity and specificity for early detection of MCI in older adults (Hobson, 2015; Julayanont and Nasreddine, 2017). According to the previous study (Lu et al., 2011) conducted in the aged Chinese population, the cutoff points used for MCI diagnosis were 13/14 for individuals with no formal education, 19/20 for individuals with 1–6 years of education, and 24/25 for individuals with 7 or more years of education. The cutoff points mentioned above were proved to be sensitive and efficient in the diagnosis of MCI in the aged Chinese population.

### Serum Parameter Measurements

Fasting blood samples were collected from all participants, and serum was separated for biochemical parameters measurement. Serum total cholesterol (TC) and triglyceride (TG) were measured using an ILAB600 clinical chemistry analyzer (Instrumentation Laboratory, Lexington, WI, United States). HDL cholesterol (HDL-c) was measured using a commercially available assay from the Instrumentation Laboratory (Lexington, WI, United States). LDL cholesterol (LDL-c) was calculated according to the Friedewald formula. All samples for each participant were analyzed within a single batch, and the interassay coefficient of variation (CV) was <5%.

### DNA Isolating and Genotyping

The Wizard Genomic DNA Purification Kit (Promega, Madison, WI, United States) was applied to extract DNA from the whole blood sample. Polymerase chain reaction (PCR) amplification and restriction fragment length polymorphism (RFLP) were used for apolipoprotein E (APOE) genotyping according to the method described by Hixson (Daydé et al., 1990). The specific primers used for APOE genotyping were forward, 5′-GGC ACG GCT GTCCAA GGA-3′ and reverse, 5′-GCC CCG GCC TGG TAC ACT GCC-3′. A total of 20% of DNA samples were genotyped again by different operators for the purpose of quality control. For APOE genotypes, participants with the E2/E2 and E2/E3 genotypes were grouped as E2 carriers; participants with E3/E4 or E4/E4 were grouped as E4 carriers; these were based on the opposite effect of E2 to that of E4 on the circulating lipid profiles. In this study, both E3/E3 and E2/E4 were classified as E3 carriers (Hixson and Vernier, 1990; Zhang et al., 2019).

### Statistics

Statistical analyses were performed using IBM SPSS v.23.0 (Chicago, IL, United States) and R v.4.0.3. Continuous variables were shown as $\bar{X}$ ± *SD*. Categorical variables were expressed as *n* (%). For numerical variables, group differences were examined using Student’s *t-*test or the paired *t*-test; for categorical variables, the chi-square test or the Mann-Whitney *U*-test was applied to assess group differences. The general linear model (GLM) was applied to analyze the difference in dietary FA intake, serum lipid profiles, and cognitive score between the two groups. For dietary FA intake, confounding factors, including BMI, physical activity, hyperlipidemia, CVA, CKD, eating fish oil supplements, and drinking alcohol, were adjusted during the analysis; for serum lipids, confounding factors, including BMI, physical activity, CVA, CKD, eating fish oil supplements, APOE genotyping, smoking, and drinking alcohol, were adjusted during the analysis; for a cognitive score, confounding factors, including AD family history, hyperlipidemia, CVA, CKD, living status, reading habit, watching TV and using the computer, BMI, smoking, drinking alcohol, and eating fish oil supplements, were adjusted during the analysis.

A binomial logistic regression was performed to analyze the impact of serum lipids and dietary FA intake on the risk of MCI in subjects with T2DM and control subjects, respectively. We categorized the participants according to the tertile of serum lipids or dietary FA intake levels into three groups (i.e., T1–T3) (Supplementary Table 1). In model 1, confounding factors including age, gender, BMI, and APOE genotype were adjusted during analysis. In model 2, potential confounding factors were further adjusted; these include smoking, drinking alcohol, physical activity, and the disease history of CVA and CKD. In model 3, education level, eating fish oil supplements, and dietary cereal, fruit, and fish intake were adjusted additionally. The odds ratio (OR) and 95% confidence interval (CI) were calculated. The statistical significance was set at *P < 0.05*.

## Results

### Demographic Characteristics and Dietary Intake of Participants

A total of 1,751 participants were recruited for the study, and 225 participants were excluded for the following reasons: an uncompleted survey questionnaire and failure to complete the whole examination (including the serum biochemical parameter measurement and APOE genotyping). Data from 1,526 subjects were used for statistical analysis. As shown in Table 1, the average age of the participants was 65.2 ± 6.2 years, and the average BMI was 25.3 ± 3.5 kg/m^2^. Men accounted for 30.4% of all participants. Among them, 377 subjects were clinically diagnosed with T2DM. There was no significant difference in age, gender, and BMI between the T2DM and control groups. Control subjects showed a higher educational level than subjects with T2DM (*P < 0.05*). The percentage of subjects with hyperlipidemia, CVA, and CKD in the T2DM group was higher than that of the control group (*P < 0.05*). In addition, subjects with T2DM had significantly lower daily cereals, fruits, and fish intake than control subjects (*P < 0.05*) (Table 2).

**TABLE 1:** Demographic characteristic of the participants.

Demographic character	T2DM (*n = 377*)	Control (*n = 1149*)	Total (*n = 1526*)	*P-*value
Age (y)^a^, $\bar{\text{X}}$ ± SD	65.7 ± 6.2	65.1 ± 6.2	65.2 ± 6.2	0.100
Gender (male)^b^, *n* (%)	129 (34.2)	335 (29.2)	464 (30.4)	0.064
BMI (kg/m^2^)^a^, $\bar{\text{X}}$± S	25.4 ± 3.3	25.3 ± 3.6	25.3 ± 3.5	0.545
**Education^c^, *n* (%)**				
Illiterate	22 (5.8)	50 (4.4)	72 (4.7)	0.036
Primary school	71 (18.8)	173 (15.1)	244 (16.0)	
Junior high school	161 (42.7)	502 (43.7)	663 (43.4)	
High school	93 (24.7)	322 (28.0)	415 (27.2)	
Junior college	20 (5.3)	67 (5.8)	87 (5.7)	
Undergraduate and above	10 (2.7)	35 (3.0)	45 (2.9)	
**Living alone^b^, *n* (%)**				
No	344 (91.2)	1068 (93.0)	1412 (92.5)	0.275
Yes	33 (8.8)	81 (7.0)	114 (7.5)	
**Reading habit^b^, *n* (%)**				
No	219 (58.1)	636 (55.4)	855 (56.0)	0.353
Yes	158 (41.9)	513 (44.6)	671 (44.0)	
**Watching TV and using the computer^b^, *n* (%)**				
No	7 (1.9)	16 (1.4)	23 (1.5)	0.521
Yes	370 (98.1)	1133 (98.6)	1503 (98.5)	
**Physical activity^c^, *n* (%)**				
*Never*	30 (8.0)	78 (6.8)	108 (7.1)	0.434
1–3 days/week	45 (11.9)	145 (12.6)	190 (12.5)	
4–6 days/week	33 (8.8)	142 (12.4)	175 (11.5)	
Everyday	269 (71.4)	784 (68.2)	1053 (68.9)	
**Smoking^c^, *n* (%)**				
No	266 (70.6)	870 (75.7)	1136 (74.4)	0.080
Abandon tobacco	52 (13.8)	108 (9.4)	160 (10.5)	
Current smoking	59 (15.6)	171 (14.9)	230 (15.1)	
**Drinking alcohol^b^, *n* (%)**				
No	269 (71.4)	839 (73.0)	1108 (72.6)	0.529
Yes	108 (28.6)	310 (27.0)	418 (27.4)	
**Diseases history^b^, *n* (%)**				
Hyperlipidemia (yes)	206 (54.6)	380 (33.1)	586 (38.4)	< 0.01
CVA (yes)	36 (9.5)	64 (5.6)	100 (6.6)	0.007
CKD (yes)	24 (6.4)	33 (2.9)	57 (3.7)	0.002
**AD family history^b^, *n* (%)**				
Yes	31 (8.2)	108 (9.4)	139 (9.1)	0.491
No	346 (91.8)	1041 (90.6)	1387 (90.9)	
**APOE genotype^b^, *n* (%)**				
E2	45 (11.9)	158 (13.8)	203 (13.3)	
E3	275 (72.9)	787 (68.5)	1062 (69.6)	0.264
E4	57 (15.1)	204 (17.8)	261 (17.1)	
**Fish oil supplements^b^, *n* (%)**				
Yes	14 (3.7)	51 (4.4)	65 (4.3)	0.809
No	351 (93.1)	1054 (91.7)	1405 (92.1)	
Missing	12 (3.2)	44 (3.8)	56 (3.7)	

*^a^Student’s t-test; ^b^Chi-square test; ^c^Mann-Whitney test. T2DM, type 2 diabetes mellitus; BMI, body mass index; AD, Alzheimer’s disease; APOE, Apolipoprotein E; CVA, cerebrovascular accident; CKD, chronic kidney disease.*

**TABLE 2:** Daily food intake in subjects with type 2 diabetes mellitus (T2DM) and control subjects.

Food item (g/d)	T2DM (*n* = 365)	Control (*n* = 365)	*P-*value
Cereals	245.82 ± 93.59	262.60 ± 98.37	0.013
Fruits	129.47 ± 99.43	161.41 ± 112.82	<0.001
Vegetables	307.88 ± 144.19	298.90 ± 136.18	0.377
Beans	27.66 ± 25.58	30.48 ± 27.58	0.149
Cooking oil	31.29 ± 20.35	29.98 ± 15.78	0.349
Fish	17.82 ± 14.70	20.92 ± 18.01	0.010
Red meat	29.40 ± 30.52	32.61 ± 31.50	0.126
Poultry	13.08 ± 12.90	14.90 ± 15.76	0.080
Nuts	17.21 ± 28.05	17.72 ± 20.94	0.778
Milk	141.48 ± 112.46	153.01 ± 91.45	0.126
Egg	32.03 ± 19.65	33.04 ± 17.42	0.448

*Data are represented as $\bar{\text{X}}$± SD. Paired t-test was used for comparison of daily food intake between subjects with T2DM with age-, gender-, and education-matched control subjects. T2DM, type 2 diabetes mellitus.*

### Cognition, Dietary Fatty Acids Intake, and Serum Lipid Levels in Type 2 Diabetes Mellitus and Control Subjects

**FIGURE 1:**
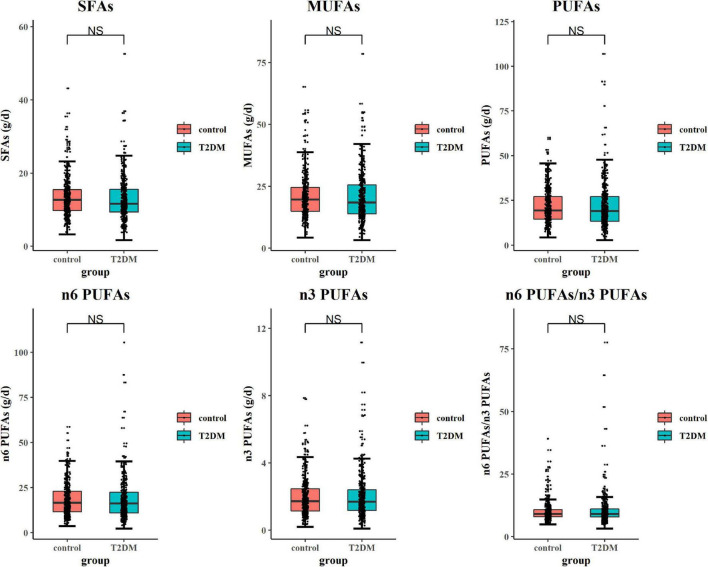
Comparison of dietary fatty acid (FA) intake in type 2 diabetes mellitus (T2DM) and control subjects. The general linear model (GLM) method was applied to analyze the difference in dietary FA intake between groups. Box plots showed each dietary FA intake in subjects with T2DM and control subjects. While comparing daily dietary FA intake, confounding factors including body mass index (BMI), physical activity, hyperlipidemia, cerebrovascular accident (CVA), chronic kidney disease (CKD), eating fish oil supplements, and drinking alcohol were adjusted.

**FIGURE 2:**
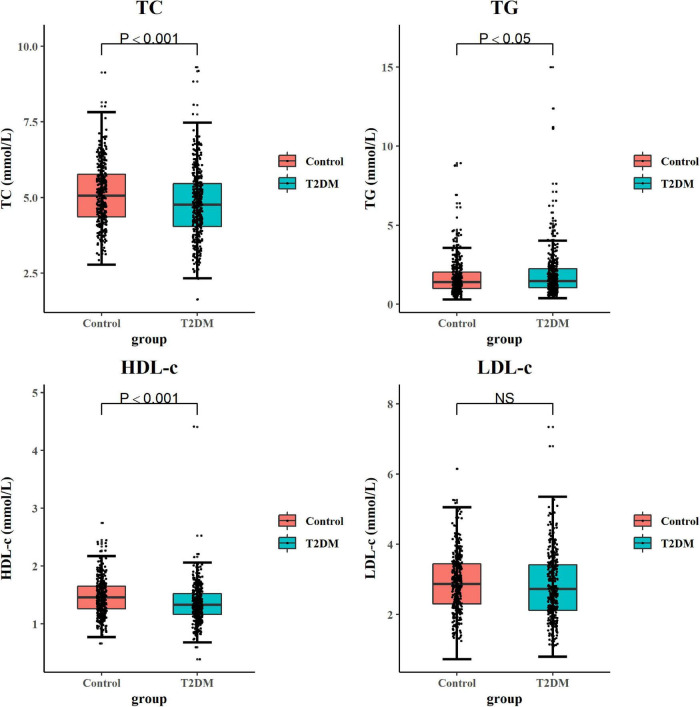
Comparison of serum lipid levels in subjects with T2DM and control subjects. The GLM method was applied to analyze the difference of serum lipids between groups. Box plots showed each serum lipid level in subjects with T2DM and control subjects. While comparing serum lipid parameters, confounding factors including BMI, physical activity, CVA, CKD, eating fish oil supplements, APOE genotype, smoking, and drinking alcohol were adjusted. Significant differences between the two groups are indicated by *P-*value (two-sided) or NS. The boxes represent the 25th, 50th (median), and 75th percentiles of the data; the whiskers show the lowest (or highest) datum. GLM, general linear model; NS, no significance; BMI, body mass index; CVA, cerebrovascular accident; CKD, chronic kidney disease; APOE, apolipoprotein.

Subjects with T2DM have better orientation abilities than the control subjects. As for other cognitive domains, there was no statistical difference between the two groups (Table 3). As shown in Figure 1, after adjusting potential confounding factors, there was no significant difference in daily dietary FAs intake between subjects with T2DM and control subjects. In comparison with control subjects, subjects with T2DM exhibited lower serum TC and HDL-c but higher serum TG levels (*P < 0.05*) (Figure 2).

**TABLE 3:** Comparison of cognitive function in subjects with type 2 diabetes mellitus (T2DM) and control subjects.

Cognition	T2DM (*n = 365*)	Control (*n = 365*)	*P-*value
Total MoCA score	24.23 ± 4.35	23.91 ± 4.94	0.35
Visual and executive	3.67 ± 1.27	3.66 ± 1.30	0.89
Naming	2.89 ± 0.38	2.88 ± 0.45	0.87
Attention	5.34 ± 1.14	5.28 ± 1.17	0.53
Language	2.03 ± 0.88	1.98 ± 0.95	0.40
Abstraction	1.51 ± 0.76	1.49 ± 0.74	0.69
Memory and delayed recall	2.73 ± 1.61	2.72 ± 1.65	0.90
Orientation	5.86 ± 0.57	5.74 ± 0.79	<0.05

*Data are expressed as mean ± SD. GLM was used to analyze the difference in cognitive score between T2DM and age-, gender-, and education-matched control groups. During data analysis, potential confounding factors including AD family history, hyperlipidemia, CVA, CKD, living status, reading habit, watching TV and using the computer, BMI, smoking, drinking alcohol, and eating fish oil supplements were adjusted. GLM, general linear model; T2DM, type 2 diabetes mellitus; MoCA, Montreal Cognitive Assessment; CVA, cerebrovascular accident; CKD, chronic kidney disease; BMI, body mass index.*

### Effects of Serum Lipid Profiles and Dietary Fatty Acids Intake on the Risk of Mild Cognitive Impairment in Subjects With Type 2 Diabetes Mellitus and Control Subjects

As shown in Table 4, after adjusting multiple confounding factors, the risk of MCI increased in subjects with T2DM with T2 or T3 levels of serum TC. There was a statistical significance between subjects with T3 level of serum TC and subjects with T1 level of serum TC (in model 1, *OR* *_T3vsT1_* = 4.65, *P* < 0.01; in model 2, *OR*_T3vsT1_ = 4.66, *P* < 0.01; and in model 3 *OR*_T3vsT1_ = 4.23, *P* = 0.016). However, the risk of MCI was decreased in subjects with T2DM with T2 (in model 1, *OR* = 0.36, *P* = 0.011; in model 2, *OR* = 0.39, *P* = 0.019; and in model 3 *OR* = 0.40, *P* = 0.033) and T3 (in model 1, *OR* = 0.24, *P* < 0.01; in model 2, *OR* = 0.23, *P* < 0.01; and in model 3 *OR* = 0.22, *P* < 0.01) levels of serum LDL-c compared with subjects in the T1 group. While in age-, gender-, and education-matched normal control group, the effect of serum TC and LDL-c levels on the risk of MCI was not found. Dietary *n*-6 PUFAs intake has no effect on the risk of MCI in subjects with T2DM. However, after further adjusting education level, fish oil supplementation, and daily cereal, fruit, and fish intake in model 3 of the control group, dietary n-6 PUFAs intake increased the risk for MCI as compared with subjects in the T1 group (*OR* *_T2vsT1_* = 2.95, *P* = 0.044; *OR* *_T3vsT1_* = 5.24, *P* = 0.042). Subjects with T2DM with T2 and T3 levels of dietary *n*-3 PUFAs intake showed the decreased risk of MCI as compared with subjects in the T1 group, and statistical significance was observed in subjects with T3 level of dietary *n*-3 PUFAs intake (in model 2, *OR* *_T3vsT1_* = 0.30, *P* = 0.044; in model 3, *OR* *_T3vsT1_* = 0.25, *P* = 0.029). In control subjects, subjects with T2 and T3 levels of dietary *n*-3 PUFAs intake showed a decreased MCI risk as compared with subjects in the T1 group, but no statistical significance was detected (*P* > 0.05). In this study, we did not find the statistically significant impact of other serum lipid parameters and dietary FA intake on the risk of MCI (Supplementary Table 2).

**TABLE 4:** Association of serum lipids level, dietary fatty acids (FAs) intake with the risk of mild cognitive impairment (MCI) in type 2 diabetes mellitus (T2DM) and control subjects.

Variable	T2DM	Model 1		Model 2		Model 3		Control	Model 1		Model 2		Model 3	
	Group	OR (95% CI)	*P*	OR (95% CI)	*P*	OR (95% CI)	*P*	Group	OR (95% CI)	*P*	OR (95% CI)	*P*	OR (95% CI)	*P*
TC (mmol/L)	T1	1.00 (Ref)	−	1.00 (Ref)	−	1.00 (Ref)	−	T1	1.00 (Ref)	−	1.00 (Ref)	−	1.00 (Ref)	−
	T2	2.12 (0.97, 4.81)	0.063	2.17 (0.97, 5.00)	0.063	2.12 (0.91, 5.11)	0.085	T2	0.98 (0.47, 2.03)	0.949	1.19 (0.55, 2.57)	0.662	1.08 (0.49, 2.43)	0.846
	T3	4.65 (1.61, 4.12)	<0.01	4.66 (1.58, 14.42)	<0.01	4.23 (1.33, 14.11)	0.016	T3	2.15 (0.83, 5.65)	0.116	2.72 (1.01, 7.47)	0.049	2.34 (0.84, 6.64)	0.106
LDL-c (mmol/L)	T1	1.00 (Ref)	−	1.00 (Ref)	−	1.00 (Ref)	−	T1	1.00 (Ref)	−	1.00 (Ref)	−	1.00 (Ref)	−
	T2	0.36 (0.16, 0.77)	0.011	0.39 (0.17, 0.84)	0.019	0.40 (0.17, 0.91)	0.033	T2	0.94 (0.47, 1.88)	0.856	0.83 (0.40, 1.71)	0.620	0.80 (0.37, 1.71)	0.574
	T3	0.24 (0.09, 0.63)	<0.01	0.23 (0.08, 0.62)	<0.01	0.22 (0.07, 0.67)	<0.01	T3	0.55 (0.23, 1.31)	0.176	0.49 (0.20, 1.21)	0.123	0.47 (0.18, 1.19)	0.113
n-6 PUFAs (g/d)	T1	1.00 (Ref)	−	1.00 (Ref)	−	1.00 (Ref)	−	T1	1.00 (Ref)	−	1.00 (Ref)	−	1.00 (Ref)	−
	T2	1.41 (0.51, 3.97)	0.511	1.57 (0.56, 4.51)	0.393	1.67 (0.54, 5.33)	0.377	T2	2.46 (0.99, 6.35)	0.058	2.60 (1.01, 6.97)	0.052	2.95 (1.05, 8.70)	0.044
	T3	3.02 (0.77, 12.13)	0.114	3.62 (0.90, 15.04)	0.073	4.62 (1.04, 21.54)	0.047	T3	4.33 (1.04, 18.87)	0.046	4.10 (0.92, 18.97)	0.066	5.24 (1.07, 26.64)	0.042
n-3 PUFAs (g/d)	T1	1.00 (Ref)	−	1.00 (Ref)	−	1.00 (Ref)	−	T1	1.00 (Ref)	−	1.00 (Ref)	−	1.00 (Ref)	−
	T2	0.51 (0.22, 1.18)	0.119	0.43 (0.18, 1.02)	0.058	0.41 (0.16, 1.05)	0.067	T2	0.67 (0.30, 1.48)	0.325	0.64 (0.28, 1.44)	0.285	0.83 (0.35, 1.95)	0.669
	T3	0.37 (0.12, 1.12)	0.082	0.30 (0.09, 0.96)	0.044	0.25 (0.07, 0.86)	0.029	T3	0.47 (0.15, 1.42)	0.186	0.48 (0.15, 1.46)	0.198	0.61 (0.18, 2.05)	0.423

*Model 1 is adjusted for age, gender, BMI, APOE genotype; Model 2 is adjusted for variables in Model 1 and smoking, drinking alcohol, physical activity, and the disease history of CVA and CKD; Model 3 is adjusted for variables in Model 2 and education level, eating fish oil supplements, dietary cereal, fruit, and fish intake. TC, total cholesterol; LDL-c, low-density lipoprotein cholesterol; PUFA, polyunsaturated fatty acid; MCI, mild cognitive impairment; T2DM, type 2 diabetes mellitus; T, tertile; Ref, reference.*

## Discussion

This study analyzed the association between serum lipids levels and dietary FA intake with cognition in subjects with T2DM and age-, gender-, and education-matched control subjects. The association between serum lipid levels and the risk of MCI was established in T2DM, but not in the control subjects. The impact of dietary FA intake on the risk for MCI was discrepant in subjects with T2DM and control subjects. These findings indicated that T2DM might affect the association of dietary FA intake or serum lipid levels with the risk for MCI in the aged Chinese subjects.

In line with the previous study (Zhen et al., 2018), we found that subjects with T2DM demonstrated an aberrant circulating lipid profile, as indicated by lower serum TC and HDL-c levels but a rather higher serum TG level in comparison with non-T2DM subjects (Figure 2). Similarly, the study of Liao et al. (2014) found that serum LDL-c and TG levels were increased, but the serum HDL-c level was decreased in patients with DM than those in control subjects. Another study reported that serum TC and LDL-c levels were significantly higher in patients with DM than those in control subjects; however, serum TC and LDL-c levels in the subjects of the well-controlled DM subgroup were significantly lower than those in the subjects of poorly controlled DM group (Erciyas et al., 2004). These results indicate that the progression of DM and the antidiabetic treatment might contribute to the observed discrepancies of circulating lipids profiles in different studies.

Dietary fat intake has been extensively reported as a regulator of circulating lipid profiles. Moreover, high dietary cholesterol and SFA intake were reported to be associated with the abnormal circulating lipid status (Kromhout and Bloemberg, 1994). Dietary intakes of rich PUFAs, especially *n*-3 FAs, and MUFAs could decrease the serum TG level and enhance the serum HDL-c level (Mensink et al., 2003). Moreover, evidence indicates that dietary PUFAs supplementation improves insulin resistance and positively affects the secretory ability of β-cells (Zubrzycki et al., 2018). However, in this study, there was no difference in dietary FA intake between T2DM and control groups (Figure 1). These data indicate that the abnormal serum lipid profile observed in subjects with T2DM could not solely be attributable to dietary FAs intake but might be explained by the status of hyperinsulinemia or abnormal endogenic lipid metabolism mediated by insulin resistance. Moreover, the contribution of dietary patterns to circulating lipid levels was also shown by other population-based studies (Thom and Lean, 2017; Kahleova et al., 2018). The health education of patients with diabetes might further promote their management of dietary patterns and dietary subgroup FA intake, which may be the possible reason for the indistinctive difference in dietary FA intake between the two groups. This was further proved by the results of the dietary survey, in which we only detected the difference in daily cereal, fruit, and fish intake between subjects with T2DM and control subjects. For other foods enriching with FAs, such as meat, poultry, nuts, egg, and cooking oil, there was no significant difference between the two groups.

In our study, serum lipid profiles showed more significant correlation with cognitive performance in the subjects with T2DM than those in the control subjects. Insulin homeostasis is important to regulate the lipolysis, but insulin resistance could disrupt and impair the lipolysis in subjects with T2DM (Petersen and Shulman, 2018). Indeed, an elevation in lipolysis was closely associated with insulin resistance; the study showed that insulin resistance did contribute to the excessive circulating FAs and the secretory profile of the adipose tissue (Morigny et al., 2016). Therefore, the increased lipolysis due to insulin resistance might further modify lipid-related cognitive decline more in individuals with T2DM than in the non-T2DM population. During the binomial logistic regression analysis, significant impacts of serum lipids (mainly TC and LDL-c) on the risk of MCI in T2DM were found in three models adjusted for multiple confounding factors. Importantly, this impact of serum lipids on the risk of MCI was not significant in age-, gender-, and education-matched control subjects. Reitz et al. (2008) study demonstrated that plasma lipid levels or lipid-lowering treatment in older adults is not associated with the risk of cognitive impairment. In contrast, a high serum TG level and a low HDL-c level were reportedly associated with MCI (Tukiainen et al., 2008). He et al. (2016) reported a higher serum TC level but a lower serum TG level in subjects with MCI than those in the control subjects. While, in our study, there was no association of the serum TG level with the risk of MCI in both subjects with T2DM and control subjects. It was suggested that a higher serum TC level might increase β-amyloid production and deposition in the brain and promote the formation of neurotoxic fibrils, thereby accelerating the progression of cognitive impairment or dementia (Qiu et al., 2009). In our study, we also detected a discrepant impact of TC on the risk of MCI in both the T2DM and the control subjects. These results might partially explain the inconsistent conclusion on the association of serum TC and cognitive function, hinting at the possible contribution of an individual’s *in vivo* pathophysiological status (such as the abnormal glucose and lipid metabolism status) to the relationship between circulating lipid parameters and the risk of MCI.

To date, the association of the serum LDL-c level with MCI and AD remains inconclusive. A cross-sectional study involving 2,000 aged Chinese subjects found that a higher serum LDL-c level was associated with a lower risk of cognitive impairment in the oldest senior adults (aged 80 years and older) but not in the younger senior adults (aged 65–79 years) (Lv et al., 2016). Studies of Chen et al. and Zou et al. reported that serum LDL-c was an independent risk factor for MCI and AD (Zou et al., 2014; Chen et al., 2019). Evidence from published literature indicates that cholesterol is essentially needed for normal neuronal functioning, and LDL-c could reduce neuronal damage and facilitate the compensatory repair of injured neurons (Sterling et al., 2016). Besides, it has been suggested that specific sub-fraction of LDL-c particles (i.e., small dense particles) are increased in patients with AD (Cagnin et al., 2007), while large and medium dense LDL-c particles showed protection against the risk of MCI (Tukiainen et al., 2012). Our data indicated that a slightly higher serum LDL-c level related to a lower risk of MCI in the aged population, especially in patients with T2DM, demonstrating that the modifying effect of insulin-resistance phenotype in subjects with T2DM might affect the association of serum LDL-c with cognition.

The impact of dietary FA intake on the risk of MCI was also demonstrated in our study. The results of a logistic regression analysis indicated that higher dietary intake of n-6 PUFAs increased the risk of MCI in both T2DM and control subjects, although, in model 1 and model 2, this impact was not statistically significant. Other studies have found that dietary components influenced cognitive decline (Solfrizzi et al., 2011). Many studies have demonstrated that fruit consumption may have a protective function on cognitive decline and that more fish consumption was suggested to be associated with a lower risk of dementia (Scarmeas et al., 2018). Indeed, in our study, daily cereals, fruits, and fish intakes were significantly lower in the subjects with T2DM than those in the control subjects. Therefore, in model 3, we further adjusted daily fish intake and fish oil supplementation, as well as fruit and cereal intakes. As expected, there was a statistically significant effect of dietary *n*-6 PUFAs intake on the risk of MCI in subjects with T2DM with T3 level of dietary *n*-6 PUFAs intake, as well as in the control subjects with T2 and T3 level of dietary *n*-6 PUFAs intake. These suggest the modifying role of dietary components on the relationship between dietary FAs intake and cognition decline. Our data indicated that, in comparison with subjects with T2DM, the cognition of non-T2DM subjects was easily affected by higher dietary *n*-6 PUFAs intake. Besides, chronic inflammation has been indicated to play a major role in the pathology of diabetes and cognition decline (Calle and Fernandez, 2012; Lontchi-Yimagou et al., 2013; Ozben and Ozben, 2019), and adherence to an anti-inflammatory diet was associated with a decreased risk for the incidence of diabetes and dementia (Martín-Peláez et al., 2020; Charisis et al., 2021). In this study, we found that patients with T2DM displayed lower daily cereals, fruits, and fish intakes. These foods were rich in anti-inflammatory components, such as vitamins E and C, polyphenols, and *n*-3 PUFAs, suggesting that a higher dietary inflammatory index of patients with T2DM might also contribute to the increased risk of MCI as compared with normal control subjects.

In this study, we only detected the protective effect of dietary *n*-3 PUFAs intake on cognition in subjects with T2DM, but no effect was observed in the control subjects. Low intake of dietary *n*-3 PUFAs is thought to be associated with increased inflammatory processes (Swanson et al., 2012), which played an important role in the pathology of diabetes (Herder et al., 2013). In our study, data from the dietary survey demonstrated that subjects with T2DM have lower fish intake than control subjects, which, together with the abnormal FA metabolism in the body caused by insulin resistance in patients with T2DM (Noll and Carpentier, 2017), might predispose the subjects with T2DM more sensitive to dietary *n*-3 PUFAs intake in comparison with non-T2DM control subjects and, therefore, strengthen the negative correlation of dietary n-3 PUFAs intake with the risk of MCI. This might partly explain the contradictory findings concerning the effects of dietary *n*-3 PUFAs intake on cognitive decline in the aged population (Martí Del Moral and Fortique, 2019). Altogether, our results indicated that the existed insulin resistance phenotype for patients with T2DM modified the relationship between dietary *n*-3 PUFAs intake and cognition.

There were some limitations existing in our study. First, instead of measuring the serum FA levels or FA concentration of the erythrocyte membrane, we only calculated dietary FA intake according to dietary survey data, which could not reflect the accurate FA level *in vivo*. Second, we failed to collect information about the usage of lipid-lowering medicine, antidiabetes treatment, and the time course of T2DM, which could influence the metabolism of FAs and lipids. These factors are potential confounding factors and should be adjusted during the data analysis in a future study. Large-scale prospective cohort studies and multiple centers randomized controlled trials are needed to provide more in-depth information to uncover the mechanisms of how diet FA intake and serum lipid levels affect cognitive performance in aged subjects with T2DM. Third, this study was a cross-sectional study; as a result, we were unable to establish the cause-effect relationship. Furthermore, cognitive decline potentially influences an individual’s daily eating behavior and food choice, especially for patients with diabetes, which might lead to a change in lipid metabolism and circulating lipid profiles (Kellar and Craft, 2020; Kullmann et al., 2020). Hence, the effect of cognition decline on the lipid profiles and FA intake in patients with T2DM need to be explored in future studies.

Collectively, our study found that, in comparison with non-T2DM aged subjects, the subjects with T2DM showed different associations of dietary FA intake and serum lipid profiles with cognition. These findings suggest that dietary and lipid intervention strategies based on an individual’s physiological (e.g., aging) and pathology statuses (e.g., T2DM) may be important for effective and precise prevention of cognitive decline in older adults.

## Data Availability Statement

The raw data supporting the conclusions of this article will be made available by the authors, without undue reservation.

## Ethics Statement

The studies involving human participants were reviewed and approved by the Medical Ethics Committee of Capital Medical University. The patients/participants provided their written informed consent to participate in this study.

## Author Contributions

LY designed the study. PL, YGa, XM, YGu, JX, XW, and NV participated in the investigation and collection of blood samples. PL performed the statistical data analysis. PL, LY, and SZ wrote the manuscript. All authors contributed to the article and approved the submitted version.

## Conflict of Interest

The authors declare that the research was conducted in the absence of any commercial or financial relationships that could be construed as a potential conflict of interest.

## Publisher’s Note

All claims expressed in this article are solely those of the authors and do not necessarily represent those of their affiliated organizations, or those of the publisher, the editors and the reviewers. Any product that may be evaluated in this article, or claim that may be made by its manufacturer, is not guaranteed or endorsed by the publisher.
