# Supplementary material for: Study on the Association of Dietary Fatty Acid Intake and Serum Lipid Profiles With Cognition in Aged Subjects With Type 2 Diabetes Mellitus
Source: Front Aging Neurosci. 2022 Mar 31;14:846132. doi: 10.3389/fnagi.2022.846132 (PMC9009143; doi:10.3389/fnagi.2022.846132)
Supplement: Supplementary file 1 [file Data_Sheet_1.docx]

**Supporting information**

**Supplementary Table 1 |** the each tertile for serum lipids level and dietary FAs intake

| Parameters | Group | Range | Parameters | Group | Range |
| --- | --- | --- | --- | --- | --- |
| TC (mmol/L) | *tertile 1* | $\leq$ 4.41 | MUFAs (g/d) | *tertile 1* | $\leq$ 16.01 |
|  | *tertile 2* | 4.42 - 5.37 |  | *tertile 2* | 16.02 - 22.17 |
|  | *tertile 3* | $\geq$ 5.38 |  | *tertile 3* | $\geq$ 22.18 |
| TG (mmol/L) | *tertile 1* | $\leq$ 1.16 | PUFAs (g/d) | *tertile 1* | $\leq$ 15.81 |
|  | *tertile 2* | 1.17 - 1.79 |  | *tertile 2* | 15.82 - 23.94 |
|  | *tertile 3* | $\geq$ 1.80 |  | *tertile 3* | $\geq$ 23.95 |
| HDL-c (mmol/L) | *tertile 1* | $\leq$ 1.26 | n-6 PUFAs (g/d) | *tertile 1* | $\leq$ 12.87 |
|  | *tertile 2* | 1.27 - 1.51 |  | *tertile 2* | 12.88 - 20.05 |
|  | *tertile 3* | $\geq$ 1.52 |  | *tertile 3* | $\geq$ 20.06 |
| LDL-c (mmol/L) | *tertile 1* | $\leq$ 2.38 | n-3 PUFAs (g/d) | *tertile 1* | $\leq$ 1.34 |
|  | *tertile 2* | 2.39 - 3.22 |  | *tertile 2* | 1.35 - 2.16 |
|  | *tertile 3* | $\geq$ 3.23 |  | *tertile 3* | $\geq$ 2.17 |
| SFAs (g/d) | *tertile 1* | $\leq$ 10.46 | n-6 : n-3 | *tertile 1* | $\leq$ 8.37 |
|  | *tertile 2* | 10.47 - 14.15 |  | *tertile 2* | 8.38 - 9.92 |
|  | *tertile 3* | $\geq$ 14.16 |  | *tertile 3* | $\geq$ 9.93 |

TC, total cholesterol; TG, triglyceride; HDL-c, high-density lipoprotein cholesterol; LDL-c, low-density lipoprotein cholesterol; SFAs, saturated fatty acids; MUFAs, monounsaturated fatty acids; PUFAs, polyunsaturated fatty acids; n-6 PUFAs, omega-6 polyunsaturated fatty acids; n-3 PUFAs, omega-3 polyunsaturated fatty acids; n-6 : n-3, the ratio of omega-6 polyunsaturated fatty acids and omega-3 polyunsaturated fatty acids.

**Supplementary Table 2** | Association between serum lipids levels, dietary FAs intake and the risk of MCI in T2DM and control subjects

| Variable | T2DM  Group | Model 1 | | Model 2 | | Model 3 | | Control  Group | Model 1 | | Model 2 | | Model 3 | |
| --- | --- | --- | --- | --- | --- | --- | --- | --- | --- | --- | --- | --- | --- | --- |
|  |  | OR (95%) | *P* | OR (95%) | *P* | OR (95%) | *P* |  | OR (95%) | *P* | OR (95%) | *P* | OR (95%) | *P* |
| TG (mmol/L) | *tertile 1* | 1.00 (Ref) | - | 1.00 (Ref) | - | 1.00 (Ref) | - | *tertile 1* | 1.00 (Ref) | - | 1.00 (Ref) | - | 1.00 (Ref) | - |
|  | *tertile 2* | 0.70 (0.37, 1.32) | 0.273 | 0.75 (0.39, 1.42) | 0.380 | 0.72 (0.36, 1.43) | 0.350 | *tertile 2* | 1.37 (0.76, 2.50) | 0.299 | 1.38 (0.75, 2.56) | 0.302 | 1.30 (0.67, 2.54) | 0.447 |
|  | *tertile 3* | 0.83 (0.45, 1.56) | 0.568 | 0.89 (0.47, 1.69) | 0.727 | 0.89 (0.45, 1.76) | 0.744 | *tertile 3* | 1.37 (0.72, 2.63) | 0.342 | 1.44 (0.74, 2.83) | 0.282 | 1.42 (0.69, 2.91) | 0.338 |
| HDL-c (mmol/L) | *tertile 1* | 1.00 (Ref) | - | 1.00 (Ref) | - | 1.00 (Ref) | - | *tertile 1* | 1.00 (Ref) | - | 1.00 (Ref) | - | 1.00 (Ref) | - |
|  | *tertile 2* | 1.07 (0.59, 1.94) | 0.819 | 1.15 (0.63, 2.12) | 0.649 | 0.99 (0.52, 1.90) | 0.986 | *tertile 2* | 1.05 (0.55, 2.03) | 0.884 | 0.97 (0.49, 1.93) | 0.941 | 0.80 (0.40, 1.64) | 0.544 |
|  | *tertile 3* | 1.76 (0.90, 3.43) | 0.097 | 1.89 (0.94, 3.79) | 0.073 | 1.87 (0.89, 3.94) | 0.098 | *tertile 3* | 1.25 (0.62, 2.54) | 0.543 | 1.13 (0.54, 2.38) | 0.745 | 1.02 (0.47, 2.26) | 0.953 |
| SFAs (g/d) | *tertile 1* | 1.00 (Ref) | - | 1.00 (Ref) | - | 1.00 (Ref) | - | *tertile 1* | 1.00 (Ref) | - | 1.00 (Ref) | - | 1.00 (Ref) | - |
|  | *tertile 2* | 0.93 (0.44, 2.00) | 0.862 | 0.93 (0.43, 2.01) | 0.854 | 1.02 (0.44, 2.37) | 0.957 | *tertile 2* | 1.02 (0.42, 2.50) | 0.969 | 1.13 (0.45, 2.87) | 0.794 | 0.81 (0.29, 2.22) | 0.674 |
|  | *tertile 3* | 0.99 (0.31, 3.16) | 0.991 | 1.06 (0.33, 3.40) | 0.927 | 1.22 (0.35, 4.29) | 0.755 | *tertile 3* | 2.40 (0.76, 7.83) | 0.141 | 2.29 (0.70, 7.70) | 0.174 | 1.22 (0.33, 4.63) | 0.765 |
| MUFAs (g/d) | *tertile 1* | 1.00 (Ref) | - | 1.00 (Ref) | - | 1.00 (Ref) | - | *tertile 1* | 1.00 (Ref) | - | 1.00 (Ref) | - | 1.00 (Ref) | - |
|  | *tertile 2* | 1.39 (0.61, 3.16) | 0.431 | 1.35 (0.58, 3.15) | 0.486 | 1.38 (0.56, 3.41) | 0.489 | *tertile 2* | 0.64 (0.26, 1.60) | 0.345 | 0.65 (0.25, 1.66) | 0.371 | 0.79 (0.28, 2.20) | 0.650 |
|  | *tertile 3* | 1.02 (0.32, 3.20) | 0.976 | 0.88 (0.27, 2.86) | 0.837 | 0.79 (0.22, 2.80) | 0.722 | *tertile 3* | 0.48 (0.13, 1.74) | 0.267 | 0.51(0.13, 1.93) | 0.327 | 0.57 (0.13, 2.41) | 0.448 |
| PUFAs (g/d) | *tertile 1* | 1.00 (Ref) | - | 1.00 (Ref) | - | 1.00 (Ref) | - | *tertile 1* | 1.00 (Ref) | - | 1.00 (Ref) | - | 1.00 (Ref) | - |
|  | *tertile 2* | 0.96 (0.41, 2.27) | 0.930 | 1.00 (0.41, 2.39) | 0.993 | 0.92 (0.34, 2.43) | 0.866 | *tertile 2* | 0.76 (0.32, 1.74) | 0.515 | 0.68 (0.28, 1.63) | 0.389 | 0.57 (0.21, 1.50) | 0.259 |
|  | *tertile 3* | 0.86 (0.27, 2.69) | 0.802 | 0.91 (0.28, 2.89) | 0.868 | 0.94 (0.26, 3.35) | 0.929 | *tertile 3* | 0.56 (0.14, 2.13) | 0.394 | 0.61 (0.15, 2.48) | 0.494 | 0.57 (0.12, 2.53) | 0.463 |
| n6 : n3 | *tertile 1* | 1.00 (Ref) | - | 1.00 (Ref) | - | 1.00 (Ref) | - | *tertile 1* | 1.00 (Ref) | - | 1.00 (Ref) | - | 1.00 (Ref) | - |
|  | *tertile 2* | 1.35 (0.74, 2.50) | 0.331 | 1.44 (0.77, 2.71) | 0.256 | 1.25 (0.64, 2.45) | 0.509 | *tertile 2* | 1.25 (0.67, 2.32) | 0.479 | 1.17 (0.61, 2.22) | 0.635 | 1.26 (0.63, 2.52) | 0.669 |
|  | *tertile 3* | 0.63 (0.30, 1.30) | 0.216 | 0.67 (0.32, 1.41) | 0.297 | 0.45 (0.20, 1.00) | 0.052 | *tertile 3* | 0.94 (0.45, 1.95) | 0.871 | 0.93 (0.44, 1.96) | 0.847 | 0.87 (0.38, 1.98) | 0.423 |

Model 1 is adjusted for age, gender, BMI, ApoE genotype; Model 2 is adjusted for variables in Model 1 and smoking, alcohol drinking, physical activity, and the disease history of CVA and CKD; Model 3 is adjusted for variables in Model 2 and education level , eating fish-oil supplement and dietary cereal, fruit and fish intake. T2DM, type 2 diabetes mellitus; TG, triglyceride; HDL-c, high-density lipoprotein cholesterol; SFAs, saturated fatty acids; MUFAs, monounsaturated fatty acids; PUFAs, polyunsaturated fatty acids; n6 : n3, the ratio of omega-6 polyunsaturated fatty acids and omega-3 polyunsaturated fatty acids; BMI, body mass index; ApoE, Apolipoprotein E; CVA, cerebrovascular accident; CKD, chronic kidney disease.
